# Shifting the center of gravity for addressing the rising cancer disease burden in Africa: A rationale for African-based integrative infectious diseases and oncology research

**DOI:** 10.1371/journal.pgph.0001970

**Published:** 2023-05-31

**Authors:** Robert F. Breiman, Georgia Demetriou, Gita Naidu, Maria A. Papathanasopoulos, Paul Ruff, Shabir A. Madhi

**Affiliations:** 1 Wits Infectious Diseases and Oncology Research Institute (IDORI), Faculty of Health Sciences, University of the Witwatersrand, Johannesburg, South Africa; 2 Division of Medical Oncology, Department of Internal Medicine, Faculty of Health Sciences, University of the Witwatersrand, Johannesburg, South Africa; 3 Paediatric Oncology Unit, Department of Paediatrics, Chris Hani Baragwanath Academic Hospital, University of the Witwatersrand, Johannesburg, South Africa; 4 HIV Pathogenesis Research Unit, Faculty of Health Sciences, University of the Witwatersrand, Johannesburg, South Africa; 5 South African Medical Research Council Common Epithelial Cancers Research Centre (CECRC), Faculty of Health Sciences, University of Witwatersrand, Johannesburg, South Africa; 6 South Africa Medical Research Council Vaccines and Infectious Diseases Analytics Research Unit, Faculty of Health Sciences, University of the Witwatersrand, Johannesburg, South Africa; McGill University, CANADA

Regardless of location, few people have escaped being touched by cancer and infectious diseases. Since the COVID-19 pandemic began, the world has a new-found respect for infectious diseases threats; people everywhere on the planet already had an awareness and substantial fear of cancer.

The relationships between infectious diseases and cancer are clear, although we have a lot to learn. Potential vaccine-mediated prevention of >90% of cervical cancers arising from the recognition of human papillomavirus (HPV) oncogenicity [1], as well as the impact of prevention and management of hepatitis B and C viral infection on dramatically reducing hepatocellular carcinoma burden [2, 3], are the most striking examples of the value of exploring such linkages. *Helicobacter pylori* and gastric cancer [4], *Schistosoma haematobium* and bladder cancer [5], Epstein-Barr virus and Burkitt’s lymphoma [6], and Human Herpesvirus 8 (HHV8) and Kaposi’s Sarcoma [7] are other notable examples.

Simplistically, infections trigger cancers through chronic inflammation of the tissues they infect, creating the potential for oncogenic mutations in those tissues, but there are multiple processes that contribute at cellular, interstitial, immunologic, vascular and molecular levels, as well as co-factors with innate host characteristics, environmental exposures, especially radiation and toxins, and potentially additional pathogens or pathogen-related substances.

Infections and oncogenesis have been characterized with a sufficient number of cancers to wonder whether we are, thus far, just scratching the surface. Upon receiving the Nobel Prize for his work on HPV and cervical cancer in 2005, Harald zur Hausen predicted more discoveries in this field—he was correct—and research efforts on infectious diseases and cancer did expand.

At least 800,000 new cancers were estimated for Africa in 2020 with >500,000 deaths, likely underestimated because of limitations in diagnostics and reporting; by 2040, 1.5 million new cases and one million deaths are projected [8]. Cancer prevention, detection, and management in sub-Saharan Africa are decades behind what exists in high-income geographies. These inequities, coupled with the substantial and rising cancer burden, serve as a clarion call for action. In response, we are building a network for sophisticated and collaborative infectious diseases and oncology research and basing it in sub-Saharan Africa. The goal of research and programs will be to produce innovative, effective, yet practical diagnostics, preventatives, and therapeutics that are designed for use in sub-Saharan Africa, and may also have global application.

Scientists in cancer and infectious diseases disciplines use sophisticated, expensive tools to advance the science. But, they typically operate in silos—clinicians and scientists in the two arenas seldom interact. We will make the case for changing that to accelerate impactful advances.

While there are an estimated 2.2 million infection-attributable cancer cases occurring each year globally [9], that figure is based on what is currently known. Why is there not a discipline of infectious oncogenesis? Perhaps if infectious disease and cancer immunologists, biologists, clinicians and epidemiologists were thinking, innovating and working together there might be unexpected and exciting discoveries bringing about an accelerated pathway towards new clinical tools. Collaborations would take such findings to the next level, developing diagnostics, prevention approaches like vaccines or novel methods, and therapies. An ideal end to end framework would include downstream processes for working with industry, advisory groups, regulatory agencies, Ministries of Health, civil society, and other stakeholders to move effective products, like early diagnostics and vaccines, into use, energizing the “discovery to action” timeline.

Are there additional infection-associated cancers that would make such a collaborative union worthwhile? To answer with a question, given that there is solid evidence for a significant relationship for six cancers already, why would we think that there are no more to be discovered using systematic approaches? There are evidence-based hypotheses to support infectious facilitation of development and outcomes for prostate, breast, colorectal cancer to name a few [10–12]. Importantly, the search for infectious disease linkages with cancer should not be limited to viruses and bacteria. The relationship between aflatoxin and liver cancer is already well described, and fungi and their products (i.e. mycotoxins) might stimulate chronic inflammation, facilitating carcinogenesis in the case of esophageal cancer [13]. The collaboration will be attentive, as well, to the potential impact of climate change on the geographic scope and magnitude of oncogenic pathogens and associated toxins, as well as the effect of climate change on access to timely diagnoses and care for infectious diseases and cancers.

It is also likely that *absence* of infectious agent(s) increases risk for certain cancers. If, for instance, the respiratory, gut, skin or other microbiome is absent a particular bacterium or diversity of bacteria, might that leave an organ prone to other carcinogenic stimuli? The diversity of organisms, especially bacteria and fungi, within tumors has been a growing topic for study [14]. Immunologic responses to infectious diseases in the context of tumor development will also need to be assessed.

What if there was an expectation of detecting infectious facilitators for a specific cancer? When a connection is shown, algorithms would be activated for a) defining the importance (i.e. what proportion of cancers in what populations are associated with the pathogen); b) developing diagnostic tests and evaluating them; c) developing therapeutics and testing them; d) developing vaccines and testing them; e) carrying out formative research and community engagement, ultimately conducting demonstration projects and forming the use cases; f) and working with industry, as well as advisory, regulatory and other policy-making bodies to advocate for and develop guidelines for use.

Benefits of an interdisciplinary approach extend beyond identifying infectious etiologies of cancers. For example, cancer and HIV research share several challenges in the development of targeted and effective therapies for eradication/cure or remission. A synergistic approach will uncover similarities between the two disciplines, and allow for the use of well-established methodologies, approaches and practices from one area to advance the other. While effective therapies for cancer may not give the desired results for HIV cure or remission, or *vice versa*, a synergistic approach will provide benefit to both disciplines.

Intentionally focusing on infectious disease and oncology linkages in academic settings in Africa is no small undertaking. However, the concept aligns with progress in shifting centers of gravity for where state-of-the-art public health research occurs [15]; sustainable progress is more credible if research to benefit populations in Africa is derived and led within the continent. Useful outcomes will require a supportive, collaborative and highly connected environment with sophisticated research investigators, laboratories and equipment, clinical research capacity, population-based community research settings and registries, as well as the capacity to translate findings into products for testing and ultimately clinical and public health use.

The University of the Witwatersrand (Wits) in Johannesburg, South Africa is establishing an Infectious Diseases and Oncology Research Institute (Wits-IDORI), built on a core of internationally recognized scientists conducting cutting edge research in cancer and infectious diseases, ranging from basic sciences to Phase III human clinical trials within well-established collaborative networks. Wits-IDORI will be a collaborative venture with scientists from universities across Africa, many already operating according to international standards, and with academic partners and industry worldwide. IDORI will engage national public health institutes, the Africa Centres for Disease Control, the African Regional Office of WHO (AFRO) and a variety of professional organizations dedicated to access to effective cancer management. Doing this work in Africa, and promoting the next generation of African scientists will help to ensure that research outputs reduce the growing burden of cancer on the continent, while strengthening scientific capacity.

Initially, Wits-IDORI will focus on priority cancers, based on burden data in Africa. Wits- IDORI will design epidemiologic, clinical, and basic science investigations to elucidate pathogen-facilitation of cancer development with an eye towards identifying prevention, diagnostic and therapeutic tools that will lead to measurable beneficial outcomes at population-levels. The Institute will also focus on research to provide evidence for minimizing devastating impacts of infectious diseases on cancer, such as optimizing timely empiric therapy for managing febrile neutropenic pediatric and adult patients.

Wits-IDORI will emphasize and promote collaboration on high quality, state of the art investigations with a culture of nurturing and retaining excellent young people in training, and junior scientists in Africa. Optimal, sustainable success of Wits-IDORI will depend on strategic investments. Wits-IDORI will build on multi-faceted scientific partnerships with African institutions, including Universities across the continent, and globally, to accelerate progress to products and strategies to reduce burden in Africa and beyond. Defining novel pathways to oncogenesis and cancer burden will pave pathways for cost-efficient diagnostics, therapies and vaccines. Public health ramifications of achieving this vision are immense.
